# Association of early failure of arteriovenous fistula with mortality in hemodialysis patients

**DOI:** 10.1038/s41598-021-85267-6

**Published:** 2021-03-11

**Authors:** Yit-Sheung Yap, Wen-Che Chi, Cheng-Hao Lin, Yi-Chun Liu, Yi-Wen Wu

**Affiliations:** 1 Adjunct Lecturer, Department of Nursing, Meiho University, No. 23, Pingguang Rd., Neipu, 912 Pingtung Taiwan, ROC; 2 Division of Nephrology, Department of Internal Medicine, Yuan’s General Hospital, Kaohsiung, 802 Taiwan, ROC; 3 Chronic Kidney Disease Education Center, Yuan’s General Hospital, Kaohsiung, 802 Taiwan, ROC

**Keywords:** Medical research, Nephrology

## Abstract

Arteriovenous fistula (AVF) is prone to early dysfunction and relates to poor outcome. However, little is known about the role of early AVF dysfunction as an independent risk factor for death in hemodialysis patients. A retrospective cohort study was performed using data of patients who underwent initial AVF surgery at a single institution. Demographic, clinical, biochemistry and AVF parameters were extracted from the electronic records, and the association between these variables and mortality was analyzed by Cox proportional hazards model. A total of 501 patients on hemodialysis (63.4 ± 12.7 years, 57.3% male) were included, and the median observation period was 3.66 years. In multivariate analysis, early failure of AVF (hazard ratio (95% confidence interval): 1.54 (1.06–2.24); p = 0.023) was associated with overall mortality but not cardiovascular mortality. Other identified predictors of overall mortality included older age, peripheral artery disease (PAD), cardiomegaly, higher white blood cell (WBC) count and corrected calcium level, and lower total cholesterol level, while predictors of cardiovascular mortality included older age, coronary artery disease (CAD), PAD and lower hemoglobin level. In conclusion, patients with early AVF failure were associated with increased risk of overall mortality.

## Introduction

Maintenance of adequate vascular access to deliver effective dialysis treatment is of vital importance for hemodialysis patients since vascular access dysfunction is associated with higher risk of mortality and morbidity^1,2^. According to the 2019 United States Renal Data System (USRDS) report, vascular access failure might impose substantial financial burden in healthcare costs due to accounts of a high percentage of hospitalizations in these hemodialysis populations^3^.

Clinical practice guidelines have recommended the arteriovenous fistula (AVF) as the first-choice access to support hemodialysis owing to good long-term patency and a low incidence of complications^4,5^. However, the AVF also has the disadvantages of higher early failure rate, which is usually caused by immaturity and thrombosis^5^. Based on previous investigations, several clinical factors have been identified as being associated with higher early failure rate of AVF, including presence of diabetes, elderly age, cardiovascular disease, etc.^6–8^. Of note, these related risk factors are also the common underlying causes of mortality in hemodialysis patients^9^.

Arteriovenous access (AVA) includes both AVF and arteriovenous graft (AVG). Recent investigations have established an association between patency loss of AVA and mortality among hemodialysis patients^10,11^. Wu et al. indicated that early patency loss of AVA (≦ 1 year) was independently associated with a higher risk of mortality in hemodialysis patients and the result was robust in patency loss of AVA of ≦ 3 months^10^. Kim et al. even demonstrated that recurrent AVA dysfunction might also predict composite all-cause mortality^11^; nevertheless, some of these studies performed analyses using only diagnostic coding and detailed information on important variables such as laboratory data was not examined, while some did not investigate the cause of death or had only an AVF subgroup in the study.

Early failure of AVF might be an important indicator of mortality among hemodialysis patients. The ability to define subsequent early AVF dysfunction is important for clinical physicians to execute more rigorous surveillance and plan interventions for these risky populations; accordingly, the main aim of this study was to investigate the association between the early failure of AVF and mortality in hemodialysis patients.

## Results

### Baseline clinical characteristics of the study patients

Table 1 shows the baseline characteristics of the study patients. A total of 501 patients whose AVF was initially created were enrolled, with a mean age of 63.4 ± 12.7 years and 57.3% were males. Among these patients, pre-existing cardiovascular diseases such as coronary artery disease (CAD), cerebrovascular disease (CVA) and peripheral artery disease (PAD) were found in 175 (34.9%), 129 (25.7%) and 53 (10.6%) patients respectively. Furthermore, 337 patients (67.3%) had cardiomegaly, while 315 patients (62.9%) were determined as cases of higher grade aortic arch calcification (AAC).

**Table 1:** Baseline characteristics of study patients according to the early failure of AVF.

Variables	Total (n = 501)	Early failure of AVF (n = 127)	Non-early failure of AVF (n = 374)	*p* value
Age (years)	63.4 ± 12.7	66.5 ± 12.3	62.4 ± 12.7	0.001*
**Gender, n (%)**				
Male	287 (57.3)	59 (46.5)	228 (61.0)	
Female	214 (42.7)	68 (53.5)	146 (39.0)	0.004*
Body mass index (kg/m^2^)	25.2 ± 5.0	25.2 ± 4.5	25.2 ± 5.2	0.885
**Comorbidities, n (%)**				
Coronary artery disease	175 (34.9)	53 (41.7)	122 (32.6)	0.063
Cerebrovascular disease	129 (25.7)	35 (27.6)	94 (25.1)	0.589
Peripheral artery disease	53 (10.6)	17 (13.4)	36 (9.6)	0.234
Diabetes mellitus	365 (72.9)	95 (74.8)	270 (72.2)	0.568
Hypertension	488 (97.4)	126 (99.2)	362 (96.8)	0.138
Hyperlipidemia	220 (43.9)	57 (44.9)	163 (43.6)	0.799
Cardiomegaly (CTR > 0.5), n (%)	337 (67.3)	88 (69.3)	249 (67.5)	0.706
**Aortic arch calcification, n (%)**				
Lower grade	182 (36.3)	22 (17.3)	160 (43.2)	
Higher grade	315 (62.9)	105 (82.7)	210 (56.8)	< 0.001*
Hepatitis C, n (%)	69 (13.8)	17 (13.8)	52 (14.7)	0.814
Hepatitis B, n (%)	48 (9.6)	13 (10.7)	35 (10.0)	0.829
**Laboratory test**				
WBC count (× 10^9^/L)	7.7 ± 2.6	8.1 ± 3.0	7.6 ± 2.5	0.098
Hemoglobin (g/L)	90.3 ± 11.8	89.5 ± 11.4	90.6 ± 11.9	0.380
Corrected calcium (mmol/L)	2.19 ± 0.19	2.21 ± 0.18	2.19 ± 0.19	0.188
Phosphorus (mmol/L)	1.88 ± 0.51	1.81 ± 0.50	1.90 ± 0.52	0.107
Serum albumin (g/L)	34.8 ± 4.6	33.6 ± 4.7	35.3 ± 4.5	< 0.001*
Total cholesterol (mmol/L)	4.46 ± 1.28	4.43 ± 1.71	4.47 ± 1.10	0.789
Triglyceride (mmol/L)	1.71 ± 1.02	1.80 ± 1.20	1.68 ± 0.96	0.285
**Medications, n (%)**				
Anti-platelet agent	182 (36.3)	51 (40.2)	131 (35.0)	0.299
ACEI/ARB	372 (74.3)	90 (70.9)	282 (75.4)	0.313
Statin/fibrate	156 (31.1)	40 (31.7)	116 (31.0)	0.878

Data are presented as mean ± standard deviation or numbers (percentages).

*AVF* arteriovenous fistula, *CTR* cardiothoracic ratio, *WBC* white blood cell, *ACEI* angiotensin converting enzyme inhibitor, *ARB* angiotensin receptor blocker.

*p < 0.05.

One hundred and twenty-seven patients (25.3%) had early failure of AVF in this study. A comparison of the clinical characteristics of early failure and non-early failure groups is also established in Table 1. Compared to the patients with non-early AVF failure, those with early AVF failure were older (p = 0.001) and predominantly female (p = 0.004), had higher grade AAC (p < 0.001), and lower levels of serum albumin (p < 0.001). There was no significant difference in comorbid conditions (CAD, CVA, PAD, diabetes mellitus, hypertension and hyperlipidemia) and medications between these two groups.

### Risk factors of overall mortality

The median observation period was 3.66 years (mean time, 4.2 ± 2.9 years), and 144 patients expired during the follow-up period. Table 2 establishes the associated variables of overall mortality by using Cox proportional hazards regression analysis. In univariate analysis, patients with early AVF failure (hazard ratio (95% confidence interval): 1.71 (1.20–2.43); p = 0.003), older age (p < 0.001), history of CAD (1.57 (1.12–2.19); p = 0.009) and PAD (2.13 (1.40–3.24); p < 0.001), presence of cardiomegaly (1.92 (1.30–2.83); p = 0.001), higher grade AAC (2.07 (1.44–2.96); p < 0.001), higher corrected calcium levels (p = 0.002) and lower phosphorus (p < 0.001) and total cholesterol levels (p = 0.006) were associated with increased risk of overall mortality. After adjustment of confounding variables, multivariate analysis showed that early failure of AVF (1.54 (1.06–2.24); p = 0.023), older age (p = 0.007), history of PAD (1.69 (1.10–2.62); p = 0.018), presence of cardiomegaly (1.67 (1.10–2.54); p = 0.017), higher white blood cell (WBC) (p = 0.037), higher corrected calcium levels (p = 0.048), and lower total cholesterol levels (p = 0.003) were associated with overall mortality.

**Table 2:** Associated factors of overall mortality among study patients by using Cox proportional hazards analysis.

Variables	Univariate		Multivariate	
	HR (95% C.I.)	p value	HR (95% C.I.)	p value
Age (per 1 year increase)	1.04 (1.02–1.05)	< 0.001*	1.02 (1.01–1.04)	0.007*
Sex (female vs male)	0.99 (0.71–1.37)	0.938		
Body mass index (per 1 kg/m^2^ increase)	0.98 (0.95–1.02)	0.302		
**Comorbidities**				
Coronary artery disease	1.57 (1.12–2.19)	0.009*		
Cerebrovascular disease	1.43 (0.99–2.07)	0.055		
Peripheral artery disease	2.13 (1.40–3.24)	< 0.001*	1.69 (1.10–2.62)	0.018*
Diabetes mellitus	1.38 (0.94–2.03)	0.105		
Hypertension	1.03 (0.38–2.78)	0.954		
Hyperlipidemia	0.91 (0.66–1.27)	0.580		
Cardiomegaly (CTR > 0.5)	1.92 (1.30–2.83)	0.001*	1.67 (1.10–2.54)	0.017*
Aortic arch calcification (lower vs higher grade)	2.07 (1.44–2.96)	< 0.001*		
Hepatitis C	1.27 (0.82–1.98)	0.284		
Hepatitis B	0.88 (0.51–1.53)	0.647		
**Laboratory test**				
WBC count (per 10^9^/L increase)	1.04 (0.97–1.10)	0.265	1.07 (1.00–1.15)	0.037*
Hemoglobin (per 10 g/L decrease)	1.05 (0.91–1.22)	0.475		
Corrected calcium (per 0.25 mmol/L increase)	1.41 (1.13–1.76)	0.002*	1.31 (1.00–1.72)	0.048*
Phosphorus (per 0.323 mmol/L decrease)	1.25 (1.11–1.41)	< 0.001*		
Serum albumin (per 10 g/L decrease)	1.37 (0.99–1.90)	0.061		
Total cholesterol (per 0.26 mmol/L decrease)	1.06 (1.02–1.11)	0.006*	1.07 (1.02–1.12)	0.003*
Triglyceride (per 0.11 mmol/L increase)	0.99 (0.98–1.01)	0.463		
**Medications**				
Anti-platelet agent	1.19 (0.85–1.66)	0.313		
ACEI/ARB	0.88 (0.62–1.24)	0.454		
Statin/fibrate	0.95 (0.66–1.35)	0.754		
Early failure of AVF	1.71 (1.20–2.43)	0.003*	1.54 (1.06–2.24)	0.023*

*CTR* cardiothoracic ratio, *WBC* white blood cell, *ACEI* angiotensin converting enzyme inhibitor, *ARB* angiotensin receptor blocker, *AVF* arteriovenous fistula.

*p < 0.05.

**Figure 1:**
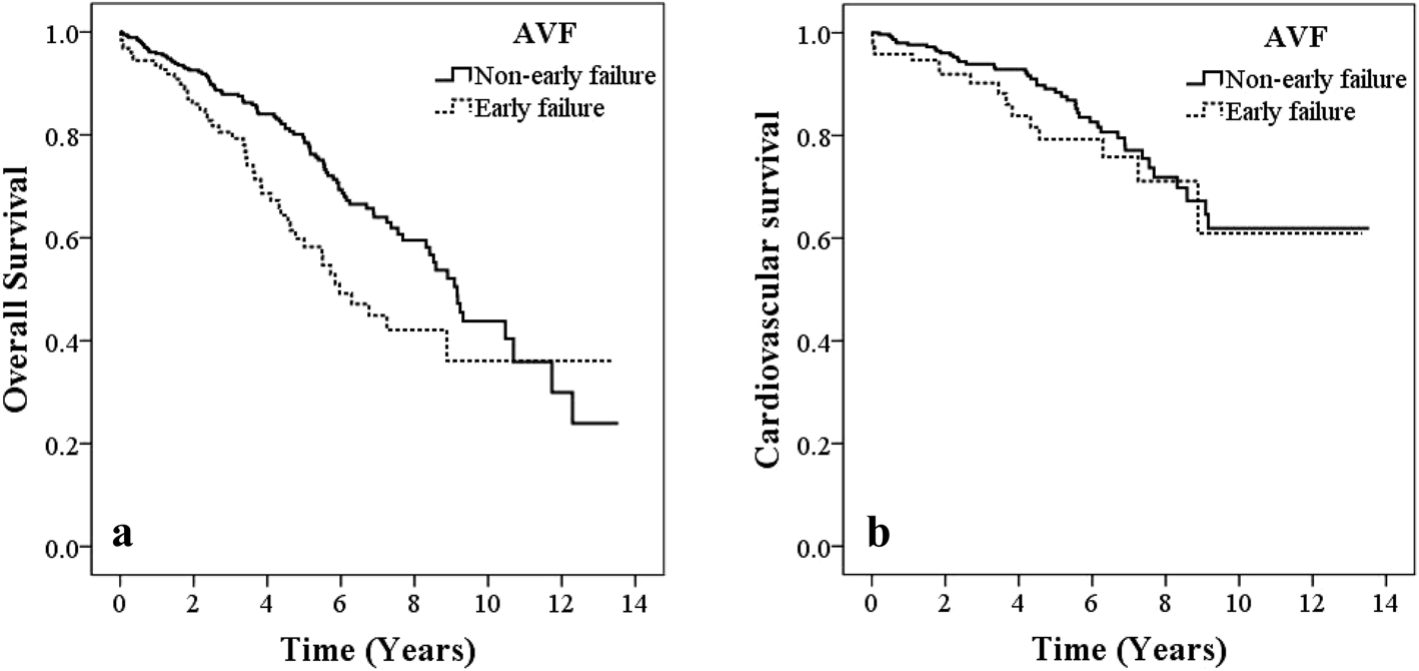
(**a**) Kaplan–Meier analyses of overall survival in patients with early failure and non-early failure of AVF. Log-rank *p* = 0.002. (**b**) Kaplan–Meier analyses of cardiovascular survival in patients with early failure and non-early failure of AVF. Log-rank *p* = 0.305.

Figure 1a reveals the Kaplan–Meier survival curves for overall survival between groups of early AVF failure and non-early AVF failure. Analysis of results showed that early failure of AVF was associated with significantly reduced overall survival than was non-early AVF failure (log-rank test; p = 0.002).

### Risk factors of cardiovascular mortality

During the follow-up period, 60 patients died of cardiovascular causes. Cox proportional hazards regression analysis of the associated variables for cardiovascular mortality is shown in Table 3. In univariate analysis, patients with older age (p = 0.011), a history of CAD (2.40 (1.44–4.00); p = 0.001) and PAD (3.09 (1.69–5.63); p < 0.001) and diabetes mellitus (2.54 (1.21–5.35); p = 0.014), presence of cardiomegaly (2.26 (1.22–4.18); p = 0.010), higher grade AAC (1.86 (1.09–3.19); p = 0.024) and lower total cholesterol level (p = 0.042) were associated with significant increase in cardiovascular mortality. In multivariate analysis, older age (p = 0.028), a history of CAD (2.15 (1.27–3.66); p = 0.005) and PAD (2.96 (1.61–5.46); p < 0.001) and lower hemoglobin level (p = 0.046) were significantly related to cardiovascular mortality. In contrast to overall mortality, early failure of AVF was not significantly related to cardiovascular mortality.

**Table 3:** Associated factors of cardiovascular mortality among study patients by using Cox proportional hazards analysis.

Variables	Univariate		Multivariate	
	HR (95% C.I.)	p value	HR (95% C.I.)	p value
Age (per 1 year increase)	1.03 (1.01–1.05)	0.011*	1.03 (1.00–1.05)	0.028*
Sex (female vs male)	1.04 (0.62–1.74)	0.881		
Body mass index (per 1 kg/m^2^ increase)	0.98 (0.92–1.03)	0.378		
**Comorbidities**				
Coronary artery disease	2.40 (1.44–4.00)	0.001*	2.15 (1.27–3.66)	0.005*
Cerebrovascular disease	1.42 (0.80–2.53)	0.233		
Peripheral artery disease	3.09 (1.69–5.63)	< 0.001*	2.96 (1.61–5.46)	< 0.001*
Diabetes mellitus	2.54 (1.21–5.35)	0.014*		
Hypertension	1.65 (0.23–11.89)	0.621		
Hyperlipidemia	1.13 (0.68–1.87)	0.644		
Cardiomegaly (CTR > 0.5)	2.26 (1.22–4.18)	0.010*		
Aortic arch calcification (lower vs higher grade)	1.86 (1.09–3.19)	0.024*		
Hepatitis C	1.02 (0.48–2.14)	0.970		
Hepatitis B	0.87 (0.37–2.02)	0.739		
**Laboratory test**				
WBC count (per 10^9^/L increase)	1.07 (0.99–1.18)	0.122		
Hemoglobin (per 10 g/L decrease)	1.22 (0.97–1.55)	0.092	1.29 (1.00–1.64)	0.046*
Corrected calcium (per 0.25 mmol/L increase)	1.26 (0.90–1.76)	0.180		
Phosphorus (per 0.323 mmol/L decrease)	1.13 (0.95–1.34)	0.183		
Serum albumin (per 10 g/L decrease)	1.47 (0.89–2.43)	0.133		
Total cholesterol (per 0.26 mmol/L decrease)	1.08 (1.00–1.15)	0.042*		
Triglyceride (per 0.11 mmol/L increase)	0.99 (0.97–1.02)	0.617		
**Medications**				
Anti-platelet agent	1.51 (0.91–2.50)	0.113		
ACEI/ARB	0.87 (0.50–1.52)	0.625		
Statin/fibrate	1.17 (0.69–1.98)	0.555		
Early failure of AVF	1.35 (0.76–2.39)	0.307		

*CTR* cardiothoracic ratio, *WBC* white blood cell, *ACEI* angiotensin converting enzyme inhibitor, *ARB* angiotensin receptor blocker, *AVF* arteriovenous fistula.

*p < 0.05.

As shown in Fig. 1b, Kaplan–Meier analysis showed that early failure of AVF was not significantly associated with reduced cardiovascular survival than was non-early failure of AVF (log-rank test; p = 0.305).

## Discussion

Compared with the general population, hemodialysis patients have an extraordinarily increased risk of mortality, and vascular access dysfunction is recognized as one of the risk factors for this^2,3^. In the present study, we demonstrated that patients with early AVF failure were associated with an increased risk of overall mortality in hemodialysis patients.

This study indicated that older and female patients were prevalent in the group of early AVF failure, and this finding is consistent with a previous study, which established that elder age had an impact on early AVF failure probably owing to higher vascular stiffness^6^. It is also postulated that smaller vessel diameters in female gender are responsible for the higher early AVF failure rate^12^. Underlining hormonal factors could also be recognized as another risk factor of early AVF failure based on prior investigation^13^. Additionally, a group of early AVF failure patients also showed higher prevalence of traditional cardiovascular risk factors like higher grade AAC. Similar findings are also seen in prior articles, in which these risk factors directly influence AVF patency^10,14^. Finally, a low serum albumin level was not only a marker of malnutrition but an inflammatory indicator as well, and might also be related to vascular access stenosis^15^.

The main finding of this study proved that patients with early AVF failure were associated with a significantly higher risk of overall but not cardiovascular mortality in comparison to patients with non-early AVF failure. Although several studies have discussed the relationship between vascular access dysfunction and mortality, the present study represents the first investigation analyzing data by using detailed records and information^10,11,16^. Kuo et al. established that dialysis vascular access dysfunction was significantly associated with an increased risk of major adverse cardiovascular events (MACE), and a significant exposure–response trend between the frequency of vascular access dysfunction and MACE was noted. Nonetheless, the parameters in the study were based on diagnostic codes and several important variables such as vascular access type, laboratory data and medications were not included in analysis since these might affect access dysfunction and cardiovascular events^16^.

Wu et al. also detected that early patency loss of AVA was independently associated with a higher risk of mortality, and they showed that patients with intervention-free intervals of < 3, 3–6, and 6–12 months had similar trends of lower survival. Similarly, the studied variables were dependent on diagnostic codes and the cause of death was not illustrated^10^. Finally, a recent publication even confirmed that recurrent AVA dysfunction also predicted cardiovascular events and composite all-cause mortality by using propensity score-matching analysis^11^.

There are several possible explanations for the links between early AVF failure and overall mortality in hemodialysis patients. Firstly, various risk factors including cardiovascular disease, presence of diabetes, malnutrition, and inflammatory state have been reported to be both main causes of the AVF dysfunction and mortality in hemodialysis patients as well. To our knowledge, these risk factors are commonly observed in dialysis populations^6,7,9,15,17^. Secondly, it is suggested that vascular disease in both arm and cardiac areas share common pathogenic mechanisms like atherosclerosis, arteriosclerosis and calcification. Based on previous findings, maturation failure of AVF might be related to combined impacts of neointimal hyperplasia and unfavorable remodeling majorly owing to preexisting calcified vessels. In fact, these vascular pathologic changes increase both AVF failure rate and mortality rate in hemodialysis patients^18,19^.

In contrast to previous studies, our results indicated that early AVF failure was not a significant risk factor for cardiovascular mortality^11,16^. It was proposed that the pathophysiology underlying the development of AVF dysfunction is complicated and multifactorial. In fact, in patients on maintenance hemodialysis, the immune disorders, chronic inflammation and endotoxemia are prevalent, and these inflammatory and endotoxin factors not only exhibit bad effect on vascular endothelial cells through causing atherogenesis but could also lead to infection status^20,21^; therefore, patients with AVF dysfunction and cardiovascular disease are also susceptible to infectious disease and other comorbidities, and these contribute to their mortality.

Consistent with previous studies, patients with older age, history of CAD, PAD and cardiomegaly were associated with an increased risk of overall and/or cardiovascular mortality^9,22,23^. In clinical settings, cardiomegaly is recognized as underlying structural cardiac disease either from left ventricular hypertrophy (LVH) or left ventricular dilatation, which could contribute to hemodialysis patients’ higher mortality rate^23^. Finally, low cholesterol and hemoglobin levels, and high WBC count and corrected calcium level, which could lead to inflammation status, atherosclerotic vascular disease and infection-related complications, might also increase risk of death among hemodialysis populations^24–26^.

The primary strength of this study was the use of a large patient population with reliable and detailed information. However, there are several potential limitations as below. Firstly, this is a single-center retrospective study; multicenter prospective studies are warranted to address this issue. Secondly, variables related to cardiovascular risk such as cardiac echogram parameters, smoking habits and inflammatory marker were not included in this study. Thirdly, the measurements for AVF patency were not completely uniform. Finally, these results were obtained from a single center and the patients were of Chinese ethnicity, so the results might not be generalizable to all hemodialysis populations.

In conclusion, we have demonstrated that early failure of AVF was significantly associated with an increased risk of overall mortality in hemodialysis patients. Patency loss of AVF might be the early indicator of mortality for patients undergoing maintenance hemodialysis; so rigorous surveillance and early treatment of these dysfunctional AVF could be beneficial in such afflicted patients.

## Methods

### Study design and participants

This retrospective cohort study was conducted using data of hemodialysis patients who had undergone first AVF from January 2006 to March 2019 at a southern regional hospital in Taiwan. For our study patients, diabetes mellitus was the most common cause of end stage renal disease (ESRD), followed by chronic glomerulonephritis. Most of these ESRD patients commenced hemodialysis due to uremic symptoms, receiving thrice-weekly hemodialysis therapy via temporary double-lumen catheter before AVF creation. The requirement for patient informed consent was waived because of the retrospective design of the study. This condition and also the study protocol were approved by the Ethics Committee of Yuan’s General Hospital (no. 20191230B), and all procedures performed involving human participants were in accordance with the 1964 Helsinki declaration.

A total of 533 hemodialysis patients who met the inclusion criteria were identified. Among these patients, those having a follow-up period of < 3 months were excluded from first AVF creation (n = 32). The remaining 501 patients were finally enrolled as study participants.

### Clinical and laboratory data collection

Patients’ baseline clinical data were collected within 3 months after first AVF placement, while laboratory parameters were extracted from routine tests (several measurements) within 3 months after initial AVF placement averaged to obtain 1 mean value. In this study, demographic and medical data including age, gender, body mass index (BMI), comorbid conditions, chest x-ray (CXR) data (assessment of AAC and cardiothoracic ratio (CTR)), concomitant medications and laboratory data were recorded; BMI was calculated as the ratio of weight (kg) divided by the square of the height (m), while CAD was defined as having a history of ischemic heart disease or undergoing percutaneous coronary angioplasty or coronary artery bypass grafts. CVA was defined as having a history of cerebral hemorrhage or stroke, while PAD included having a history of this disease, undergoing percutaneous angioplasty or endarterectomy of peripheral vessels, or an ankle–brachial index ≤ 0.9.

All laboratory values were performed by our hospital laboratory using automated and standardized methods. The following laboratory parameters were assessed: anti-HCV (S/CO), HBsAg (IU/ml), WBC count (10^9^/L), hemoglobin (g/L), phosphorus (mmol/L), corrected calcium (mmol/L), serum albumin (g/L), total cholesterol (mmol/L), and triglyceride (mmol/L). Hepatitis C infection was defined as anti-HCV titer ≧ 1.00 S/CO, while Hepatitis B infection was defined as HBsAg titer ≧ 0.05 IU/ml. Otherwise, corrected serum calcium (mg/dL) was defined as observed serum calcium (mg/dL) + [0.8 × (4 − serum albumin) (g/dL)], if the serum albumin was  less than 4 g/dL.

### Evaluation of aortic arch calcification and cardiothoracic ratio

CXR were assessed for the presence of AAC using a specific scale. Each CXR was reviewed by two physicians blinded to patient conditions. Briefly, AAC extent was divided into four grades according to the following categorization: grade 0, no visible calcification; grade 1, small spots of calcification or single thin calcification of the aortic knob; grade 2, one or more areas of thick calcification, but ≤ 50% of the circular area of the aortic knob; grade 3, circular calcification with > 50% of circular area of the aortic knob^27^. Grades 0–1 and grades 2–3 were defined as lower and higher AAC grade respectively. Additionally, the CTR was defined as the ratio of a maximum diameter of the cardiac to the maximum diameter of the thorax on CXR, with cardiomegaly defined as CTR > 50%.

### Arteriovenous fistula dysfunction and outcome measurement

With respect to vascular access, ‘fistula first’ policy was carried out and as was percutaneous transluminal angioplasty, being the major method to treat vascular access dysfunction in our institution. Before vascular access placement, preoperative sonographic vascular mapping was performed in a large portion of our patients for the purpose to identify vessels suitable for access creation. The decision regarding type and location of the initial access creation was individually based on clinical finding and surgeons’ opinion. Generally, a forearm fistula was created preferentially, but upper arm fistulas or grafts were placed when the forearm vessels were inadequate. Finally, permanent central venous catheter placement was the last resort option for patients with poor vessel condition.

Early AVF failure was defined as an AVF that never developed to the point that it could be used or failed within the first 3 months of usage after access operation^19^. It mainly comprised immaturity and thrombosis in this study, which was consistent with prior studies^5,19^. Patency of AVF was regularly monitored by physical examination and Transonic machine (measurement of access flow every 3 months) after access creation. The conditions for AVFs that failed to develop adequately to support dialysis or failed within 3 months of usage were defined as the following: inadequate AVF blood flow, occlusion, elevated venous pressure, difficult cannulation and limited cannulation site (such as deep, small or stiff vessels causing shorter length of the cannulation site), other complications (such as easily ruptured vessels causing puncture hematoma) leading to nonfunctional access, and were referred for percutaneous transluminal angioplasty or revision surgery.

The primary and secondary outcomes of interest were time to overall and cardiovascular mortality respectively. Information on cardiovascular death was garnered from the electronic medical records, and was defined as death from CAD, congestive heart failure, fatal arrhythmia and stroke. For mortality analyses, patients remained at risk until death, censoring for kidney transplantation or shifting to peritoneal dialysis, transferal to another dialysis clinic, unavailability for follow-up, or end of the study period (31 October 2019).

### Statistical analysis

All data are presented as frequencies with percentages for categorical variables, mean values with standard deviation for continuous variables. Comparison between groups (early failure versus non-early failure) was performed by the Chi-square test, Fisher exact test and independent samples *t* test as appropriate.

Survival curves of overall and cardiovascular mortality were estimated by Kaplan–Meier analysis while groups between early failure and non-early failure were compared by the log-rank test. Then, univariate and multivariate (stepwise method) Cox proportional hazard models were used to estimate the independent predictors of overall and cardiovascular mortality in all patients. Test results were presented as hazard ratios (HR) with 95% confidence intervals (CI), and two-sided *p* < 0.05 was considered statistically significant. The SPSS statistical software version 19 (SPSS Inc., Chicago, IL, USA) was used in all descriptive and outcome analyses.

### Statement of ethics

The study protocol was approved by the Ethics Committee of Yuan’s General Hospital, Taiwan (no. 20191230B), and all procedures performed involving human participants were in accordance with the 1964 Helsinki declaration. The requirement for informed patient consent was waived given the retrospective nature of the study and minimal participant risk.

## References

[1] Feldman HI, Kobrin S, Wasserstein A (1996). Hemodialysis vascular access morbidity. J. Am. Soc. Nephrol..

[2] Polkinghorne KR, McDonald SP, Atkins RC, Kerr PG (2004). Vascular access and all-cause mortality: A propensity score analysis. J. Am. Soc. Nephrol..

[3] United States Renal Data System. 2019 USRDS Annual Data Report: Epidemiology of kidney disease in the United States. National Institutes of Health, National Institute of Diabetes and Digestive and Kidney Diseases, Bethesda, MD, 2019. https://www.usrds.org/annual-data-report/current-adr/. Accessed 3 Nov 2020.

[4] Vascular Access 2006 Work Group (2006). Clinical practice guidelines for vascular access. Am J Kidney Dis..

[5] Tordoir J, Canaud B, Haage P, Konner K, Basci A, Fouque D (2007). EBPG on vascular access. Nephrol. Dial. Transpl..

[6] Abreu R, Rioja S, Vallespin J, Vinuesa X, Iglesias R, Ibeas J (2018). Predictors of early failure and secondary patency in native arteriovenous fistulas for hemodialysis. Int. Angiol..

[7] Smith GE, Gohil R, Chetter IC (2012). Factors affecting the patency of arteriovenous fistulas for dialysis access. J. Vasc. Surg..

[8] Ernandez T, Saudan P, Berney T, Merminod T, Bednarkiewicz M, Martin PY (2005). Risk factors for early failure of native arteriovenous fistulas. Nephron. Clin. Pract..

[9] Ma L, Zhao S (2017). Risk factors for mortality in patients undergoing hemodialysis: A systematic review and meta-analysis. Int. J. Cardiol..

[10] Wu CK, Lin CH, Hsu CC, Tarng DC, Kor CT, Chen YC (2018). Association of early loss of primary functional patency of arteriovenous access with mortality in incident hemodialysis patients: A nationwide population-based observational study. Medicine (Baltimore)..

[11] Kim HJ, Lee H, Kim DK, Oh KH, Kim YS, Ahn C (2016). Recurrent vascular access dysfunction as a novel marker of cardiovascular outcome and mortality in hemodialysis patients. Am. J. Nephrol..

[12] Pounds LL, Teodorescu VJ (2013). Chronic kidney disease and dialysis access in women. J. Vasc. Surg..

[13] Miller CD, Robbin ML, Allon M (2003). Gender differences in outcomes of arteriovenous fistulas in hemodialysis patients. Kidney Int..

[14] Yap YS, Ting KT, Chi WC, Lin CH, Liu YC, Chuang WL (2016). Aortic arch calcification predicts patency loss of arteriovenous fistula in end-stage renal disease patients. Sci. Rep..

[15] Ocak G, Rotmans JI, Vossen CY, Rosendaal FR, Krediet RT, Boeschoten EW (2013). Type of arteriovenous vascular access and association with patency and mortality. BMC Nephrol..

[16] Kuo TH, Tseng CT, Lin WH, Chao JY, Wang WM, Li CY (2015). Association between vascular access dysfunction and subsequent major adverse cardiovascular events in patients on hemodialysis: A population-based nested case-control study. Medicine (Baltimore)..

[17] Monroy-Cuadros M, Yilmaz S, Salazar-Banuelos A, Doig C (2010). Risk factors associated with patency loss of hemodialysis vascular access within 6 months. Clin. J. Am. Soc. Nephrol..

[18] Lee JY, Kim YO (2017). Pre-existing arterial pathologic changes affecting arteriovenous fistula patency and cardiovascular mortality in hemodialysis patients. Korean J. Intern. Med..

[19] Asif A, Roy-Chaudhury P, Beathard GA (2006). Early arteriovenous fistula failure: A logical proposal for when and how to intervene. Clin. J. Am. Soc. Nephrol..

[20] Akchurin OM, Kaskel F (2015). Update on inflammation in chronic kidney disease. Blood Purif..

[21] Wong J, Vilar E, Farrington K (2015). Endotoxemia in end-stage kidney disease. Semin. Dial..

[22] Yang Y, Ning Y, Shang W, Luo R, Li L, Guo S (2016). Association of peripheral arterial disease with all-cause and cardiovascular mortality in hemodialysis patients: A meta-analysis. BMC Nephrol..

[23] Stack AG, Serna H, Ramsanahie A, Henry C (2004). Determinants and prognostic importance of cardiomegaly among new ESRD patients in the United States. Ann. Epidemiol..

[24] Rivara MB, Ravel V, Kalantar-Zadeh K, Streja E, Lau WL, Nissenson AR (2015). Uncorrected and albumin-corrected calcium, phosphorus, and mortality in patients undergoing maintenance dialysis. J. Am. Soc. Nephrol..

[25] Chmielewski M, Verduijn M, Drechsler C, Lindholm B, Stenvinkel P, Rutkowski B (2011). Low cholesterol in dialysis patients-causal factor for mortality or an effect of confounding?. Nephrol. Dial. Transpl..

[26] Kuo KL, Hung SC, Tseng WC, Tsai MT, Liu JS, Lin MH (2018). Association of anemia and iron parameters with mortality among patients undergoing prevalent hemodialysis in Taiwan: The AIM—HD study. J. Am. Heart Assoc..

[27] Symeonidis G, Papanas N, Giannakis I, Mavridis G, Lakasas G, Kyriakidis G (2002). Gravity of aortic arch calcification as evaluated in adult Greek patients. Int. Angiol..

